# Development of Immunoassays for Foodborne Pathogenic Bacteria Detection Using PolyHRP for Signal Enhancement

**DOI:** 10.3390/bios15050318

**Published:** 2025-05-15

**Authors:** Yijia Zhang, Junkang Pan, Qiyi He, Zhihao Xu, Bruce D. Hammock, Dongyang Li

**Affiliations:** 1 College of Biosystems Engineering and Food Science, Zhejiang University, Hangzhou 310058, China; 22413026@zju.edu.cn (Y.Z.); 12413042@zju.edu.cn (J.P.); qiyhe@zju.edu.cn (Q.H.); zhihaoxu@zju.edu.cn (Z.X.); 2 Zhejiang Key Laboratory of Intelligent Sensing and Robotics for Agriculture, Zhejiang University, Hangzhou 310058, China; 3 Department of Entomology and Nematology and UCD Comprehensive Cancer Center, University of California Davis, Davis, CA 95616, USA; bdhammock@ucdavis.edu

**Keywords:** PolyHRP, foodborne pathogenic bacteria, immunoassay, signal enhancement

## Abstract

The rapid and accurate detection of foodborne pathogens is essential for ensuring food safety. *Escherichia coli* O157:H7 (*E. coli* O157:H7) and *Salmonella Typhimurium* (*S. Typhimurium*) are major foodborne pathogenic bacteria that pose significant public health risks, highlighting the need for effective detection methods. In this study, highly sensitive double-antibody sandwich-based enzyme-linked immunosorbent assays (ELISAs) were developed for the rapid detection of *E. coli* O157:H7 and *S. Typhimurium*, utilizing a streptavidin-polymerized horseradish peroxidase (SA-PolyHRP)-based signal enhancement system. Systematic optimization was performed on key parameters, including the capture antibody concentration, detection antibody, and blocking agent. Compared to the method using SA-HRP, substitution with SA-PolyHRP significantly improved detection sensitivity, achieving limits of detection (LODs) of 1.4 × 10^4^ CFU/mL for *E. coli* O157:H7 and 6.0 × 10^3^ CFU/mL for *S. Typhimurium*, with sensitivity enhancements of 7.86-fold and 1.83-fold, respectively. Specificity tests confirmed no cross-reactivity with non-target or closely related pathogenic strains. The matrix effect was effectively mitigated through 10-fold and 100-fold dilutions for *E. coli* O157:H7 and *S. Typhimurium*, respectively. Both pathogens were successfully detected in beef samples spiked with 5 CFU after 5 h of incubation. This study demonstrates the effectiveness of PolyHRP-based signal enhancement for the highly sensitive and specific detection of foodborne pathogens, offering a promising approach for rapid food safety monitoring and public health protection.

## 1. Introduction

The rapid and accurate detection of foodborne pathogens is a crucial component for ensuring food safety and optimizing the control of microbial contamination during food processing and storage. The public health and socioeconomic hazards caused by foodborne pathogens have become a global challenge [1,2]. According to data released by the World Health Organization (WHO) in 2015, approximately 600 million people worldwide fall ill annually due to consuming microbiologically contaminated food, resulting in 420,000 deaths and causing the loss of 33 million healthy life years, with nearly 29% of cases directly attributed to contaminated food [3,4]. The World Bank 2022 study further stated that the total productivity losses associated with foodborne illnesses amount to approximately USD 95.2 billion per year, while medical expenditure totals around USD 15 billion per year [5]. Bacteria, as the second leading cause of foodborne diseases, pose particularly significant risks, with approximately 3.6 million cases of bacterial foodborne illnesses occurring annually in the USA, of which 1.5 million cases are attributed to diarrheagenic *E. coli*, *Salmonella*, and *Shigella* [6]. *Salmonella* remains one of the top causative agents, with 3086 foodborne disease outbreaks and 20,017 human cases reported in the 27 European Union (EU) member states in 2020 [7]. Among the various bacterial foodborne pathogens, *E. coli* O157:H7 and *S. Typhimurium* pose particularly severe threats. As a Shiga toxin-producing strain (STEC), *E. coli* O157:H7 exhibits an exceptionally low infectious dose (minimum infectious dose of 10–100 bacteria), capable of inducing a spectrum of severe illnesses ranging from diarrhea to hemorrhagic colitis and hemolytic uremic syndrome, with potential progression to renal failure or death [8]. It accounts for 20% of foodborne diseases globally and 73,000 cases annually in the USA [9,10]. Although *S. Typhimurium* has a high threshold of infection (>10^5^ CFU/mL), it is widely found in animal-derived food products such as meat, poultry, eggs, and dairy products, enabling clinical manifestations ranging from enteritis and typhoid fever to systemic infections, which is the primary causative agent of foodborne outbreaks in the EU and low- and middle-income countries [7,11,12]. Both of these pathogens continue to threaten the safety of the food supply chain and public health by virtue of their low infectious dose and high risk of contamination [13].

The gold standard for detecting foodborne pathogens is culture methods, which are time-consuming and not suitable for situations requiring rapid testing [14]. Immunoassay has become an important tool for the rapid detection of pathogenic bacteria due to its simplicity and low cost [15,16,17,18]. There are various immunoassay detection methods for foodborne pathogens, including ELISA, lateral bow immunoassay (LFIA), chemiluminescent immunoassay (CLIA), time-resolved fluorescent immunoassay (TRFIA), and biosensors [19,20].

Among the various immunoassay methods available, ELISA is the most widely used technique due to its great advantages in terms of sensitivity, speed, simplicity, cost, throughput, and safety [21,22]. However, its conventional colorimetric mode relies on alkaline phosphatase and HRP-catalyzed color development, exhibiting a relatively low sensitivity [23]. Therefore, improving the sensitivity of traditional colorimetric ELISA for the detection of foodborne pathogens has become a current research focus and hotspot. The biotin–streptavidin system, while serving as a classical strategy to enhance immunoassay sensitivity by augmenting enzyme payload, achieves only limited sensitivity gains despite its capability for signal enhancement [24]. Recently, technology for coupling numerous HRPs to SA has attracted attention as an alternative to the traditional biotin–streptavidin system. Mishra et al. utilized a biotinylated secondary antibody and SA-PolyHRP, which resulted in a 110-fold increase in Western blotting sensitivity over the conventional method [25]. The higher the molar ratio of HRP to streptavidin, the more HRP molecules react with the substrate per binding. The higher the molar ratio of HRP to streptavidin, the more HRP molecules react with the substrate during each binding event. Consequently, compared to conventional SA-HRP conjugates, SA-PolyHRP generates a more pronounced signal enhancement effect without requiring additional procedural steps [26,27].

**Scheme 1:**
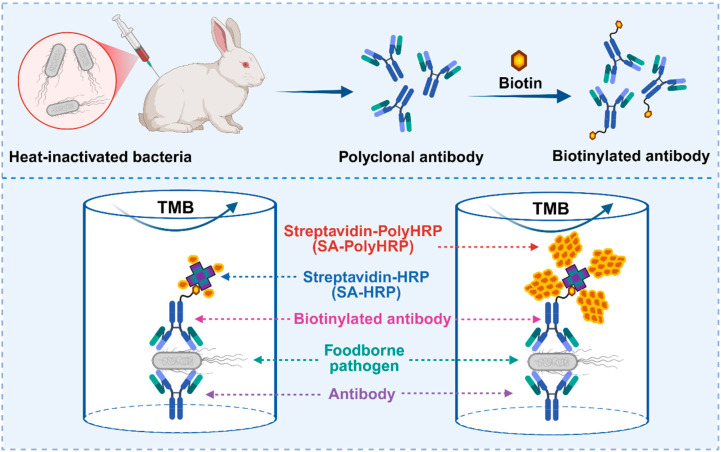
Schematic comparison of sandwich ELISA formats for foodborne pathogenic bacteria detection by PolyHRP and HRP.

In this study, we developed double-antibody sandwich-based ELISAs for the detection of two foodborne pathogenic bacteria using SA-PolyHRP as a signal-amplifying tracer (Scheme 1). In order to optimize the experimental parameters, we systematically investigated the types and concentrations of the capture antibody, detection antibody, and blocking agent. Furthermore, double-antibody sandwich ELISAs based on SA-PolyHRP were developed and their accuracy was evaluated across medium matrices, successfully detecting *E. coli* O157:H7 in beef samples. These findings underscore the potential of SA-PolyHRP as a signal amplification tool in foodborne pathogen monitoring.

## 2. Experimental Section

### 2.1. Materials

*E. coli* O157:H7 (ATCC 35150), *S. Typhimurium* (ATCC 14028), *Listeria monocytogenes* (ATCC 19111), *Vibrio parahaemolyticus* (ATCC 17802), and *Staphylococcus aureus* (ATCC 6538) were purchased from Huankai Microbial (Guangzhou, China). Other common *E. coli* engineered strains were gifts from Dr. Suqing Zhao’s lab at Guangdong University of Technology. The SA-HRP, 3,3′,5,5′-tetramethybenzidine (TMB), and Sulfo-NHS-LC-biotin were purchased from Sigma-Aldrich. SA-PolyHRP, produced by Stereospecific Detection Technologies (SDT) GmbH (Baesweiler, Germany), was purchased from Fitzgerald Industries International (Concord, MA, USA). Nunc MaxiSorp high-binding flat-bottom 96-well plates were used in our experiment and purchased from Thermo Fisher Scientific Inc. (Rockford, IL, USA). A Bio Tek 405 TS 96-channel plate washer (Aglient, Santa Clara, CA, USA) was used for plate washing. Absorbance at 450 nm was recorded by BioTek Epoch 2 (Aglient). Skim milk was purchased from EMD Millipore, Burlington, MA, USA. TMB substrate solution and PBS buffer were prepared as described in our previous work [27]. Bovine serum albumin (BSA) was purchased from Sangon Biotech (Shanghai, China). Modified buffered peptone water with pyruvate medium (mBPWp) was purchased from Hopebio (Shandong, China). All reagents were of analytical grade unless otherwise stated.

### 2.2. Preparation of Polyclonal Antibody Against Foodborne Pathogenic Bacteria

Referring to previous studies, female New Zealand rabbits were selected to generate specific polyclonal antibodies against foodborne pathogenic bacteria. For the initial immunization, 1 mL of *E. coli* O157:H7 and *S. Typhimurium* (1 × 10^8^ CFU/mL) was emulsified with an equal volume of Freund’s complete adjuvant and administered via multiple subcutaneous injections into the cervical and dorsal regions of the rabbits. Fourteen days after the initial immunization, blood was collected, and antibody-containing serum was isolated. This was followed by four booster immunizations using Freund’s incomplete adjuvant, with blood collection performed one week after each injection. The serum antibody titer was evaluated using ELISA. Finally, polyclonal antibodies were purified from the rabbit serum using a protein A agarose affinity chromatography column.

### 2.3. Biotinylation of Anti-E. coli O157:H7 pAb and Anti-S. Typhimurium pAb

The anti-*E. coli* O157:H7 pAb and anti-*S. Typhimurium* pAb were biotinylated using Sulfo-NHS-LC-Biotin via amine coupling, with a molar ratio of biotin to pAb of 10:1. Fresh Sulfo-NHS-LC-Biotin solution (10 mg/mL in PBS) was rapidly added to 1 mL of anti-*E. coli* O157:H7 pAb or anti-*S. Typhimurium* pAb solution. The reaction mixture was then incubated for 1 h at room temperature with gentle shaking. To remove unreacted biotin, the resulting solution was dialyzed (MWCO of 3K) with 1 L of PBS for 24 h at 4 °C and replaced three times. The biotinylated anti-*E. coli* O157:H7 pAb and biotinylated anti-*S. Typhimurium* pAb obtained were stored at −20 °C before use.

### 2.4. Optimization of a Double-Antibody Sandwich-Based ELISA for E. coli O157:H7

A double-antibody sandwich-based ELISA system was established with anti-*E. coli* O157:H7 polyclonal antibody (pAb) as the capture antibody and biotinylated anti-*E. coli* O157:H7 pAb as the detection antibody. To optimize the blocking conditions, the blocking efficiencies of different agents were systematically evaluated, including 3% and 5% (*w*/*v*) skim milk, as well as 3% and 5% (*w*/*v*) BSA, all prepared in PBS. Following the determination of 5% (*w*/*v*) skim milk in PBS as the optimal blocking agent, single-factor optimization experiments were conducted to screen the optimal concentrations of the capture and detection antibodies. The concentration gradients of the antibodies were set at 0.25, 0.5, 1, 2, 4, and 8 μg/mL for both the capture and detection antibodies. All experiments were performed in triplicate (*n* = 3) to ensure data reproducibility and accuracy.

### 2.5. Optimization of a Double-Antibody Sandwich-Based ELISA for S. Typhimurium

The optimization method of the *S. Typhimurium* double-antibody sandwich ELISA followed the same procedure as that in Section 2.4.

### 2.6. Development of Double-Antibody Sandwich-Based Immunoassays

Based on the optimization results from Section 2.4 and Section 2.5, the capture antibodies were selected. In this assay, microplates were coated with 100 μL/well of the pAb and incubated overnight at 4 °C. After washing with PBST, the wells were subsequently blocked with 5% (*w*/*v*) skim milk in PBS (270 μL/well) for 1 h. After another washing step, serial dilutions of *E. coli* O157:H7 or *S. Typhimurium* (100 μL/well, PBS) were introduced and incubated. Subsequently, 100 μL/well of biotinylated anti-*E. coli* O157:H7 pAb or biotinylated anti-*S. Typhimurium* pAb diluted with 3% (*w*/*v*) skim milk in PBS was added separately and incubated for 1 h. The wells were then washed again, and 100 μL/well of SA-PolyHRP (1:40,000 dilution, 25 ng/mL, PBS containing 5% BSA) was added and incubated for 1 h for comparison with the SA-HRP (1:10,000 dilution, 100 ng/mL, PBS containing 5% BSA). After the final wash, 100 μL/well of TMB substrate was added for colorimetric detection, allowing color development to proceed for 10 min. The reaction was terminated by the addition of 50 μL/well of sulfuric acid, and the optical density (OD) was measured at 450 nm.

### 2.7. Specificity

A cross-reactivity analysis was conducted to evaluate the specificity of the proposed methods. To verify this specificity, foodborne pathogenic bacteria, including *S. Typhimurium*, *Listeria monocytogenes*, *Vibrio parahaemolyticus*, *Staphylococcus aureus*, and other *E. coli* strains, were also subjected to cross-reactivity testing in the proposed ELISA.

### 2.8. Matrix Effect

To evaluate the accuracy and reliability of the proposed ELISA method in a variety of sample matrices, spiking recovery assays were performed to investigate the matrix effects for *E. coli* O157:H7 and *S. Typhimurium*. Predefined bacterial concentrations of *E. coli* O157:H7 and *S. Typhimurium* were spiked into mBPWp medium at serial dilutions (undiluted, 1:10 dilution, and 1:100 dilution) to simulate varying matrix complexities. The spiked samples were subsequently analyzed using the optimized ELISA protocol to quantify recovery rates and evaluate matrix-induced signal suppression or enhancement.

### 2.9. Analysis of Food Samples

The proposed method was used to evaluate the practicality of the developed ELISA by analyzing food samples of beef, as well as samples spiked with 5 CFU of *E. coli* O157:H7 and *S. Typhimurium*. Sample preparation and enrichment were performed in accordance with the procedures specified [8]. These samples were subjected to a plate counting method after enrichment. The rest of the samples were then heat-inactivated and diluted to different concentrations for subsequent ELISA analysis.

## 3. Result and Discussion

### 3.1. Characterization of the Anti-E. coli O157:H7 and Anti-S. Typhimurium pAb

**Figure 1:**
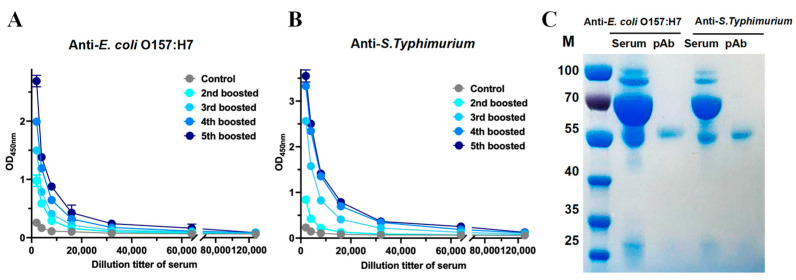
Characterization of anti-*E. coli* O157:H7 and anti-*S. Typhimurium* serum and pAb for antibody preparation. Serum titer of two rabbits separately immunized by *E. coli* O157:H7 (**A**) and *S. Typhimurium* (**B**) to ensure successful acquisition of antibody sources. (**C**) Characterization of anti-*E. coli* O157:H7 and anti-*S. Typhimurium* serum and purified pAb by SDS-PAGE.

Reportedly, the performance and specificity of an immunoassay often depend on the successful preparation of antibodies. To ensure that the antibodies obtained through immunization met the desired performance criteria, serum from the rabbits was obtained after the second round of immunization and tested for potency. As shown in Figure 1A,B, both rabbits exhibited robust immune responses to the target organisms. The determination of serum titer was based on the fold dilution method, specified as the maximum dilution in the serum dilution series at which the OD_450nm_ intensity can reach 2.1 times the signal intensity of the negative control. Following five rounds of immunization, the serum achieved a titer of 1:128,000, demonstrating that a substantial quantity of antibodies was successfully generated from the rabbit serum. The purification of pAb was performed using Protein A affinity chromatography. The SDS-PAGE electrophoresis results (Figure 1C) revealed two prominent bands at 55 kDa and 25 kDa in the purified antibody, indicating a significantly reduced heterogeneity compared to the original serum. This confirms that the obtained pAb was of high purity and suitable for subsequent experiments.

### 3.2. Optimization for E. coli O157:H7 and S. Typhimurium Sandwich-Based ELISA

**Figure 2:**
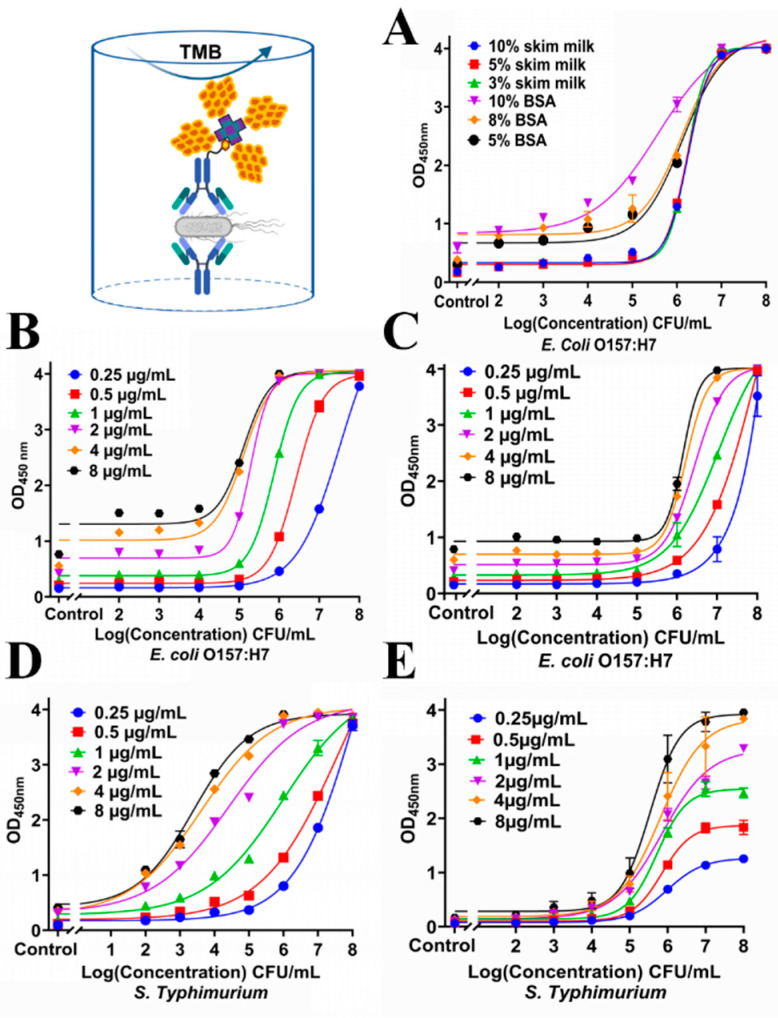
Optimization curves for *E. coli* O157:H7 and *S. Typhimurium* sandwich ELISA systems for blocking agent and antibody working concentration. (**A**) Optimization of blocking agent. (**B**) Optimization of *E. coli* O157:H7 capture antibody. (**C**) Optimization of *E. coli* O157:H7 detection antibody. (**D**) Optimization of *S. Typhimurium* capture antibody. (**E**) Optimization of *S. Typhimurium* detection antibody.

Through the systematic optimization of the detection systems for *E. coli* O157:H7 and *S. Typhimurium*, the optimal concentrations of capture antibodies, detection antibodies, and type and concentration of the blocking agent were determined (Figure 2). To evaluate the effect of blocking agents on the PolyHRP-based reaction system, three concentration gradients of two blocking agents were compared (Figure 2A). The results demonstrated that 5% (*w*/*v*) skim milk in PBS significantly reduced nonspecific binding signals and achieved signal enhancement by PolyHRP with a high OD_450nm_ at the same time. For the detection of *E. coli* O157:H7 (Figure 2B,C), 1 μg/mL of anti-*E. coli* O157:H7 pAb was used as the capture antibody, paired with 1 μg/mL of biotinylated anti-*E. coli* O157:H7 pAb as the detection antibody. Under these working concentrations, the performance of the assay achieved good positive/negative ratios while efficiently minimizing antibody consumption. Detailed P/N ratio data of the optimization across different pathogenic bacteria concentrations have been incorporated into Tables S1–S4. As for *S. Typhimurium* (Figure 2D,E), the assay utilized 2 μg/mL of anti-*S. Typhimurium* pAb as the capture antibody and 2 μg/mL of biotinylated anti-*S. Typhimurium* pAb as the detection antibody. Consequently, in order to obtain parameters with lower background noise and a higher sensitivity, an efficient double-antibody sandwich ELISA was established.

### 3.3. Double-Antibody Sandwich-Based ELISA for E. coli O157:H7 and S. Typhimurium

**Figure 3:**
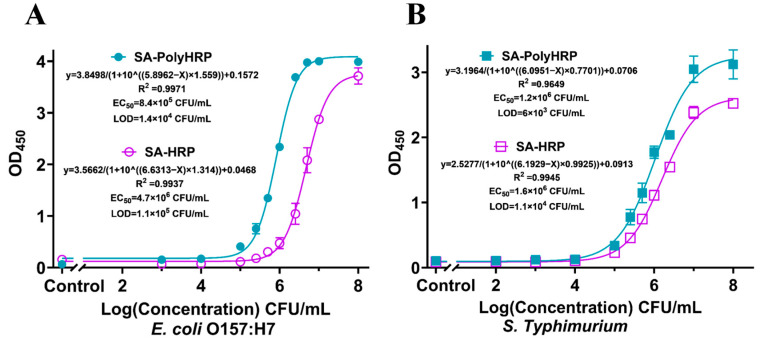
The quantification curves of *E. coli* O157:H7 (**A**) and *S. Typhimurium* (**B**) were detected by SA-PolyHRP and SA-HRP under the same conditions based on double-antibody sandwich ELISA, respectively. Error bars indicate standard deviations (*n* = 3).

The double-antibody sandwich-based ELISA was established according to the results of the parameters optimized in Section 3.2, with the standard curves of *E. coli* O157:H7 and *S. Typhimurium* constructed, respectively (Figure 3). The sensitivities of SA-PolyHRP and SA-HRP as signal amplification systems were compared. The experimental results demonstrated a significant correlation between the absorbance at 450 nm and the logarithmic concentration of the target bacteria, which was effectively modeled using a four-parameter logistic equation for both the SA-PolyHRP and SA-HRP detection systems. With the model fitting, the regression equations for SA-PolyHRP and SA-HRP for *E. coli* O157:H7 were y = 3.8498/(1 + 10^((5.8962 − x) × 1.559)) + 0.1572 (R^2^ = 0.9971) and y = 3.5662/(1 + 10^((6.6313 − x) × 1.314)) + 0.0468 (R^2^ = 0.9937). For *S. Typhimurium*, the equations were y = 3.1964/(1 + 10^((6.0951 − x) × 0.7701)) + 0.0706 (R^2^ = 0.9649) and y = 2.5277/(1 + 10^((6.1929 − x) × 0.9925)) + 0.0913 (R^2^ = 0.9945) for SA-PolyHRP and SA-HRP, respectively, where y represents the absorbance at 450 nm and x denotes the logarithmic concentration of the target bacteria (CFU/mL). The LOD was calculated based on the bacterial concentration corresponding to the mean absorbance of blank samples plus three times the standard deviation. For *E. coli* O157:H7 and *S. Typhimurium*, the LODs of the SA-PolyHRP system were 1.4 × 10^4^ CFU/mL and 6.0 × 10^3^ CFU/mL, respectively, indicating 7.86-fold and 1.83-fold higher results than those achieved with the SA-HRP system. Further analysis of the half-maximal effective concentration (EC_50_) demonstrated that the SA-PolyHRP system achieved 5.6-fold higher results for *E. coli* O157:H7 and 1.3-fold higher results for *S. Typhimurium* than those of the SA-HRP system, consistent with the LOD results. These findings indicate that the SA-PolyHRP system significantly outperformed the conventional SA-HRP system in the detection of *E. coli* O157:H7 and *S. Typhimurium*, collectively validating its enhanced capability in signal enhancement and sensitivity improvement. This superior performance is likely attributable to the higher enzyme-loading capacity of the PolyHRP complex. This optimized strategy provides a reliable methodological foundation for the high-sensitivity detection of foodborne pathogens.

### 3.4. Cross-Reactivity

**Figure 4:**
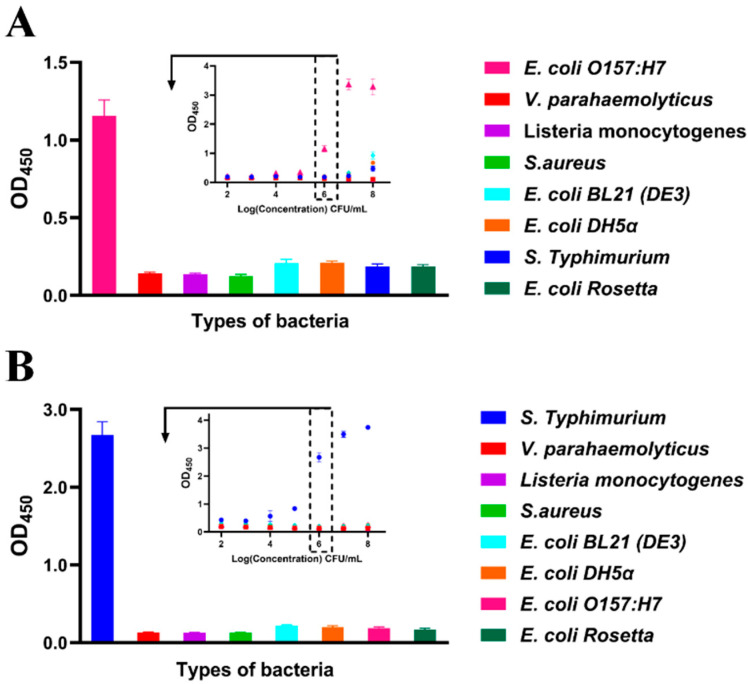
The specificity assay of SA-PolyHRP-based double-antibody sandwich ELISA for *E. coli* O157:H7 (**A**) and *S. Typhimurium* (**B**) compared with other foodborne pathogens and *E. coli* at 10^6^ CFU/mL, respectively. Insert shows standard curves (from 10^2^ to 10^8^ CFU/mL) for the detection of *E. coli* O157:H7 (**A**) or *S. Typhimurium* (**B**) and other pathogenic bacteria and *E. coli* strains. Error bars indicate standard deviations (*n* = 3).

To validate the specificity of the double-antibody sandwich-based immunoassay, cross-reactivity evaluations were systematically conducted for both the *E. coli* O157:H7 and *S. Typhimurium* detection systems. The detection targets encompassed other strains of *E. coli*, as well as common foodborne pathogens, including *Listeria monocytogenes*, *Vibrio parahaemolyticus*, and *Staphylococcus aureus*. Additionally, *E. coli* O157:H7 and *S. Typhimurium* were tested separately against each other for cross-reactivity. As shown in Figure 4, both the *E. coli* O157:H7 and *S. Typhimurium* detection systems exhibited exclusive specificity at 10^6^ CFU/mL, demonstrating target-specific responses with no detectable cross-reactivity toward non-target bacterial strains, including phylogenetically related species and clinically relevant foodborne pathogens. This study demonstrates the potential application of the pAb sandwich-based ELISA for the detection of two foodborne pathogens.

### 3.5. Spike-and-Recovery Analysis

To evaluate the matrix effect and validate the effectiveness of the proposed method, a spike-and-recovery analysis was conducted in mBPWp medium. As pre-enrichment steps are commonly employed in practical detection to enhance sensitivity, the matrix effect of the enrichment broth was further investigated. As shown in Table 1, the recovery rates for *E. coli* O157:H7 were 115–139% (undiluted), 84–99% (1:10 dilution), and 74–97% (1:100 dilution), while those for *S. Typhimurium* were 121–194% (undiluted), 109–165% (1:10 dilution), and 93–130% (1:100 dilution). The results demonstrated that increasing the dilution factor effectively reduced the matrix effect. For *E. coli* O157:H7 detection, a dilution factor of 1:10 or higher is recommended, whereas for *S. Typhimurium*, a dilution factor of 1:100 or higher is advised to ensure that recovery rates remain within acceptable ranges, thereby validating the applicability and reliability of the method.

### 3.6. Analysis Performance of Food Samples Detection

To validate the practicality of the proposed method, we conducted spiked sample tests on beef with different enrichment timepoints (Table 2). The experimental results showed that after enrichment treatment, the target bacteria were not detected in samples that were not intentionally spiked. When spiked with 5 CFU of bacteria and incubated for 5 h, the target bacteria could be detected in beef samples using both ELISA and the plate counting method. Furthermore, as the incubation time increased, both the ELISA and plate counting results demonstrated a corresponding upward trend in bacterial concentration. Notably, although ELISA data were generally higher than those obtained from plate counting, we hypothesize that this discrepancy may have arisen from the fact that plate counting exclusively measures viable bacteria, whereas this study implemented heat-inactivation treatment prior to testing to mitigate potential handling risks. Consequently, the number of inactivated bacteria in the samples would be expected to exceed the number of viable bacteria, providing a plausible explanation for the observed differences.

## 4. Conclusions

In summary, we successfully developed a highly sensitive detection method based on a double-antibody sandwich ELISA using SA-PolyHRP to achieve signal amplification for the rapid detection of *E. coli* O157:H7 and *S. Typhimurium*. Through the systematic optimization of key parameters, including capture antibodies, detection antibodies, and blocking agents, a stable detection system was established. Compared with the method using SA-HRP, the replacement with SA-PolyHRP significantly enhanced detection sensitivity, with LODs reaching 1.4 × 10^4^ CFU/mL and 6.0 × 10^3^ CFU/mL for the two target pathogens, corresponding to sensitivity improvements of 7.86-fold and 1.83-fold, respectively. As shown in Table 3, we analyzed the LODs of typical immunoassays for the detection of *E. coli* O157:H7 and *S. Typhimurium*, which revealed the advantages of using PolyHRP signal enhancement for our assay. Although numerous ultrasensitive detection techniques (such as nanomaterial-enhanced lateral flow assays and electrochemical immunoassays) can achieve lower limits of detection, these methods are rarely adopted in practical applications. Particularly for some nanomaterials, their reproducibility and stability still require further validation in commercial applications. In contrast, the PolyHRP used in this study is a mature, commercially available reagent that is readily accessible to any research group or company. Our findings demonstrate that the mere introduction of PolyHRP significantly enhances detection performance. This discovery provides critical insights for both research and practical applications of the ELISA-based detection of foodborne pathogenic bacteria.

At the same time, we also found that although PolyHRP provided a stronger signal output, its non-specific binding was also enhanced. Therefore, in addition to the basic blocking steps, unlike traditional ELISA, it is necessary to add extra blocking agents later in the process to effectively reduce non-specific adsorption. We need to strike a balance between high-specificity binding and low nonspecific binding to better improve sensitivity. Our specificity experiments demonstrated that the method exhibited no cross-reactivity with non-target strains or closely related pathogens, indicating an excellent selectivity. The matrix effect of the method was investigated in enrichment broths, with the results for *E. coli* O157:H7 and *S. Typhimurium* showing near elimination of the matrix effect by 10-fold and 100-fold dilution, respectively. Furthermore, the contents of the target pathogens in beef samples with an increase in the enrichment timepoint and the additional spiking of 5 CFU were also successfully tested, and the results were compared with those obtained with the plate counting method. The developed SA-PolyHRP-based immunoassay can be a cost-effective solution for routine high-throughput pathogen screening in different food supply chains, especially in resource-limited settings. Overall, this study provides a simple and universal signal amplification strategy for the highly sensitive and specific detection of foodborne pathogens, which has important potential applications in food safety monitoring and public health prevention and control.

## Figures and Tables

**Table 1:** Spike-and-recovery analysis of *E. coli* O157:H7 and *S. Typhimurium* in spiked mBPWp medium by a sandwich immunoassay.

Target	Spiked (CFU/mL)	Undiluted mBPWp Medium Founded (CFU/mL)	Recovery (%)	1:10 Dilution Founded (CFU/mL)	Recovery (%)	1:100 Dilution Founded (CFU/mL)	Recovery (%)
*E. coli* O157:H7	1.25 × 10^6^	1.4 × 10^6^ ± 1.3 × 10^5^	115%	1.2 × 10^6^ ± 1.0 × 10^5^	93%	1.2 × 10^6^ ± 1.1 × 10^4^	97%
	6.25 × 10^5^	8.7 × 10^5^ ± 3.4 × 10^4^	139%	6.2 × 10^5^ ± 9.7 × 10^4^	99%	5.6 × 10^5^ ± 2.1 × 10^4^	90%
	3.125 × 10^5^	3.8 × 10^5^ ± 2.9 × 10^3^	122%	2.6 × 10^5^ ± 4.5 × 10^4^	84%	2.3 × 10^5^ ± 1.3 × 10^4^	74%
*S. Typhimurium*	1.25 × 10^6^	1.5 × 10^6^ ± 2.5 × 10^4^	121%	1.4 × 10^6^ ± 5.6 × 10^4^	109%	1.2 × 10^6^ ± 7.5 × 10^4^	93%
	6.25 × 10^5^	1.0 × 10^6^ ± 8.6 × 10^3^	162%	9.3 × 10^5^ ± 5.6 × 10^4^	150%	7.3 × 10^5^ ± 7.7 × 10^4^	116%
	3.125 × 10^5^	6.0 × 10^5^ ± 3.1 × 10^4^	194%	5.2 × 10^5^ ± 1.8 × 10^4^	165%	4.1 × 10^5^ ± 2.6 × 10^4^	130%

**Table 2:** A comparison of sandwich ELISA and plate counting test for the analysis of *E. coli* O157:H7 and *S. Typhimurium* in beef samples.

Target		Control ^c^		Spiked with 5 CFU	
	Enrichment Period	ELISA ^d^ (CFU/mL)	Plate Counting (CFU/mL)	ELISA (CFU/mL)	Plate Counting ^e^ (CFU/mL)
*E. coli* O157:H7 ^a^	5 h	N.D.	N.D.	2.8 × 10^8^ ± 3.0 × 10^6^	2.3 × 10^8^
	18 h	N.D.	N.D.	9.2 × 10^8^ ± 5.5 × 10^7^	7.7 × 10^8^
*S. Typhimurium* ^b^	5 h	N.D.	N.D.	8.2 × 10^8^ ± 1.3 × 10^8^	1.9 × 10^6^
	18 h	N.D.	N.D.	3.5 × 10^10^ ± 2.0 × 10^9^	2.2 × 10^10^

^a^ For *E. coli* O157:H7 amplification, 25 g of beef was added to 225 mL of mBPWp medium. ^b^ For *S. Typhimurium* enrichment, we added 25 g of beef to 225 mL of LB medium and incubated at 37 °C. ^c^ The control samples were incubated at 37 °C for 5 h, then add 1 mL each of the supplements per 225 mL of mBPWp medium and incubated at 42 °C overnight for 18 h. ^d^ Results are average ± SD (CV, *n* = 3). All samples were diluted in 10^2^, 10^3^, and 10^4^ with sterile PBS for ELISA; and 10^3^–10^11^ fold dilution with sterile PBS for plate counting tests. ^e^ Data on *E. coli* O157:H7 were from ref. [5].

**Table 3:** Application of immunoassays for detection of *E. coli* O157:H7 and *S. Typhimurium*.

Target	Principle	Combined Technique	LOD ^a^ (CFU/mL)	Sample	Reference
*E. coli* O157:H7	ELISA	PolyHRP	1.4 × 10^4^	Beef	This work
	ELISA	Core–shell red silica nanoparticles	4.5 × 10^5^	Milk and pork	[28]
	Lateral flow immunoassay	Gold superparticles added to polymer nanobead	5.59 × 10^2^	Milk	[29]
	Lateral flow immunoassay	Aggregation-induced emission material labeling antigen as a fluorescent probe	10^5^	Beef, milk, and fruits	[30]
*S. Typhimurium*	ELISA	PolyHRP	6.0 × 10^3^	Beef	This work
	ELISA	Nb-based ELISA	9.15 × 10^3^	milk, honey, pork, and lettuce	[22]
	ELISA	Phage-mediated double-nanobody sandwich chemiluminescent enzyme immunoassay	3.63 × 10^3^	Juice, honey, milk, and pork	[31]
	ELISA	Sandwich ELISA	1.25 × 10^6^	Milk	[32]
	ELISA	IMS-ELISA	10^4^	Chicken	[33]

^a^ The LOD values were extracted from method establishment phases (standard curve calibration) in the cited literature.

## Data Availability

Data will be made available on request.

## Supplementary Materials

The following supporting information can be downloaded at https://www.mdpi.com/article/10.3390/bios15050318/s1, Table S1: Correlation analysis between *E. coli* O157:H7 concentration and capture antibody concentrations based on signal-to-noise ratio (P/N); Table S2: Correlation analysis between *E. coli* O157:H7 concentration and detection antibody concentrations based on signal-to-noise ratio (P/N); Table S3: Correlation analysis between *S. Typhimurium* concentration and capture antibody concentrations based on signal-to-noise ratio (P/N); Table S4: Correlation analysis between *S. Typhimurium* concentration and detection antibody concentrations based on signal-to-noise ratio (P/N).
