# Supplementary material for: Development of Immunoassays for Foodborne Pathogenic Bacteria Detection Using PolyHRP for Signal Enhancement
Source: Biosensors (Basel). 2025 May 15;15(5):318. doi: 10.3390/bios15050318 (PMC12109803; doi:10.3390/bios15050318)
Supplement: Supplementary file 1 [file biosensors-15-00318-s001.zip › biosensors-3574862-supplementary.pdf]

biosensors

MDPI

Supplementary

Development of Immunoassays for Foodborne Pathogenic Bacteria Detection Using PolyHRP for Signal Enhancement

Yijia Zhang [1,2], Junkang Pan [1,2], Qiyi He [1,2], Zhihao Xu [1,2], Bruce D. Hammock [3] and Dongyang Li [1,2,*]

[1] College of Biosystems Engineering and Food Science, Zhejiang University, Hangzhou 310058, China; 22413026@zju.edu.cn (Y.Z.); 12413042@zju.edu.cn (J.P.); qiyhe@zju.edu.cn (Q.H.); zhihaoxu@zju.edu.cn (Z.X.)
[2] Zhejiang Key Laboratory of Intelligent Sensing and Robotics for Agriculture, Zhejiang University, Hangzhou 310058, China
[3] Department of Entomology and Nematology and UCD Comprehensive Cancer Center, University of California Davis, Davis, CA 95616, USA; bdhammock@ucdavis.edu
* Correspondence: dylee@zju.edu.cn

Table S1. Correlation analysis between *E. coli* O157:H7 concentration and capture antibody concentrations based on signal-to-noise ratio (P/N).

Log (Concentration)	Concentration of *E. coli* O157:H7 capture antibody (μg/mL)					
CFU/mL	0.25	0.5	1	2	4	8
8	over range	over range	over range	over range	over range	over range
7	10.2 ± 0.1	16.3 ± 1.1	over range	over range	over range	over range
6	3.0 ± 0.0	5.1 ± 0.3	**8.8 ± 0.1**	over range	over range	over range
5	1.3 ± 0.0	1.5 ± 0.0	**2.1 ± 0.1**	3.4 ± 0.0	4.0 ± 0.0	3.2 ± 0.1
4	1.1 ± 0.0	1.1 ± 0.0	**1.4 ± 0.1**	2.0 ± 0.0	2.4 ± 0.0	2.1 ± 0.1
3	1.1 ± 0.0	1.3 ± 0.1	**1.4 ± 0.1**	1.8 ± 0.1	2.2 ± 0.0	2.0 ± 0.1
2	1.1 ± 0.1	1.2 ± 0.0	**1.4 ± 0.0**	1.9 ± 0.0	2.1 ± 0.0	2.0 ± 0.1

Table S2. Correlation analysis between *E. coli* O157:H7 concentration and detection antibody concentrations based on signal-to-noise ratio (P/N).

Log (Concentration)	Concentration of *E. coli* O157:H7 detection antibody (μg/mL)					
CFU/mL	0.25	0.5	1	2	4	8
**8**	over range	over range	over range	over range	over range	over range
**7**	5.2 ± 1.3	7.5 ± 0.2	**8.2 ± 0.4**	over range	over range	over range
**6**	2.3 ± 0.3	2.8 ± 0.3	**3.5 ± 0.5**	3.3 ± 0.0	2.9 ± 0.1	2.5 ± 0.2
**5**	1.3 ± 0.0	1.4 ± 0.1	**1.4 ± 0.1**	1.5 ± 0.0	1.3 ± 0.0	1.2 ± 0.0
**4**	1.2 ± 0.1	1.3 ± 0.0	**1.2 ± 0.1**	1.4 ± 0.1	1.2 ± 0.0	1.2 ± 0.0
**3**	1.0 ± 0.0	1.1 ± 0.1	**1.2 ± 0.1**	1.3 ± 0.0	1.2 ± 0.0	1.2 ± 0.0
**2**	1.0 ± 0.1	1.2 ± 0.1	**1.2 ± 0.0**	1.3 ± 0.0	1.3 ± 0.0	1.3 ± 0.0

Table S3. Correlation analysis between *S. Typhimurium* concentration and capture antibody concentrations based on signal-to-noise ratio (P/N).

Log (Concentration)	Concentration of *S. Typhimurium* capture antibody (μg/mL)					
CFU/mL	0.25	0.5	1	2	4	8
8	38.1 ± 0.4	32.4 ± 0.4	20.2 ± 1.0	over range	over range	over range
7	19.1 ± 1.1	21.1 ± 0.8	17.4 ± 1.4	over range	over range	over range
6	8.2 ± 0.2	11.4 ± 0.4	12.9 ± 0.5	over range	over range	over range
5	3.8 ± 0.2	5.5 ± 0.0	6.8 ± 0.1	**7.8 ± 0.7**	9.0 ± 0.7	8.4 ± 0.4
4	3.4 ± 0.3	4.5 ± 0.1	5.2 ± 0.3	**6.4 ± 0.3**	7.3 ± 0.3	6.9 ± 0.6
3	2.4 ± 0.2	2.9 ± 0.1	3.1 ± 0.1	**3.8 ± 0.4**	4.4 ± 0.1	4.0 ± 0.6
2	1.8 ± 0.2	2.0 ± 0.1	2.3 ± 0.1	**2.5 ± 0.4**	2.9 ± 0.1	2.6 ± 0.2

Table S4. Correlation analysis between *S. Typhimurium* concentration and detection antibody concentrations based on signal-to-noise ratio (P/N).

Log (Concentration)	Concentration of *S. Typhimurium* detection antibody (μg/mL)					
CFU/mL	0.25	0.5	1	2	4	8
8	20.1 ± 0.2	29.3 ± 2.4	30.9 ± 1.1	**32.3 ± 0.3**	over range	over range
7	18.2 ± 1.1	29.2 ± 1.8	31.8 ± 1.7	**26.1 ± 1.1**	over range	over range
6	11.1 ± 0.9	18.3 ± 1.2	21.8 ± 1.1	**20.3 ± 1.1**	17.8 ± 3.0	17.7 ± 1.8
5	3.2 ± 0.1	4.7 ± 0.1	6.0 ± 0.0	**6.3 ± 0.1**	5.9 ± 1.0	5.7 ± 0.7
4	1.9 ± 0.0	2.5 ± 0.0	3.0 ± 0.1	**3.5 ± 0.1**	2.9 ± 0.6	2.8 ± 0.4
3	1.5 ± 0.0	2.0 ± 0.1	2.1 ± 0.1	**2.4 ± 0.1**	2.0 ± 0.3	2.1 ± 0.3
2	1.2 ± 0.1	1.4 ± 0.1	1.4 ± 0.0	**1.5 ± 0.0**	1.3 ± 0.0	1.3 ± 0.1
